# Penology

**Published:** 1880-10

**Authors:** J. B. Chandler


### "PENOLOGY."

J. B. CHANDLER.

How few among us, have given this subject the least thought, yet it is one of the utmost importance in its bearings upon the welfare of all communities.

With the advantage of intimate personal relations with one, now at rest, who devoted a large portion of a useful life to the close study of Penology, its relations to both society and prisoners, the writer of this has treasured a few thoughts which may prove of interest to some of the readers of the BISTOURY.

Penology embraces within its study not only Webster's definition, "The science which treats of public punishments, as they respect the public and the sufferer," but the causes which lead to, and the effects which follow the punishments.

In the space to be spared in a journal like the BISTOURY to any subject, but little, comparatively, can be said upon any one branch, yet we may touch lightly on some prominent points and perhaps do more in a future number.

CRIME CAUSE is essentially a vital part of this subject. Why does the prisoner suffer punishment? Why did he commit an outrage upon society which led to imprisonment? Some crimes result from deep laid schemes, plans which have occupied time in developing; others from the outburst of ungovernable passions; but be they what they may, petty larceny or wilful murder, or any of the many phases of crime which lie between the extremes, nearly, if not entirely, all are traceable directly to vicious or imperfect early training.

The lack of proper parental care, indeed the utter carelessness as to the early training of youth too frequently manifested. Fathers closely interested in getting the daily bread or so occupied in schemes for the acquiring of wealth that no time can be given to their children; mothers either constantly burdened with the demands for attention to new comers to the flock, thus deprived from doing their duty towards the elder children, or utterly careless what the mischief may be, so the noise and damage done is not at home. Indeed, the temper manifested in a babe, unchecked may be the germ of a passion which may in after life lead to murder. The prisoner who in parting with his mother at the gallows bit off her ear, seemed to have realized that to her neglect he owed his crime and its punishment.

The influences of home, the cultivation of family affections, the respect for the feelings of others, the deference to age, are all acknowledged elements of good, but how sadly are they neglected even amongst the educated and cultivated classes. Whilst our country is being peopled largely by those whose circumstances are such that scarcely a roof can be kept over large and increasing families and the struggle for food is the all-absorbing matter of interest, children live in the streets and know home only as a roosting place. From this class, is it any wonder that most of the prisons are filled ?

Another view of Penology opens up a field for thought, "To what purpose are criminals imprisoned, and will punishment prevent a recurrence of crime when the term of the sentence has been complied with ?"

Of course whilst the convict is in his cell he can commit no depredations on society, except so far as forcing the taxpayers to support him. But turn him loose on the world merely punished by a temporary curtailment of his liberty, with no effort to eradicate early impressions which led to crime, and is it likely that he will not return to vicious ways, with a more studious avoidance of the folly which brought about detection ?

Many good men throughout the world just now are busy with this problem and applying experimental remedies.

Again, the advances in civilization have possibly led to the adoption of measures of mercy, which protect the hardened and vicious from a severity of punishment which might deter some from a constant repetition of crime. Convictions for the same offence by a single individual have been as frequent as forty times! These lead to demands for the restoration of chain gang labor and the whipping post in some communities.

Our subject grows upon us and in touching merely upon its heads, upon any one of which a volume might be profitably written, we but offer our readers food for elaborate thought. As the population becomes denser crime increases and the remedy for it must interest all.

The work must begin at the cradle; guard the threshold, train the child, engraft upon its religious training practical views of *meum* and *tuum*, not only as far as stealing is concerned, but upon all relations of life. Let him realize even from a selfish point of view, that honesty, respectfulness and general propriety will always produce the most happiness to that community in which they are fully exercised, and thus reflectively the individual must be happier.

